# Supplementary material for: Efficacy of Ultrasound-Guided Percutaneous Lavage and Biocompatible Electrical Neurostimulation, in Calcific Rotator Cuff Tendinopathy and Shoulder Pain, A Prospective Pilot Study
Source: Int J Environ Res Public Health. 2022 May 11;19(10):5837. doi: 10.3390/ijerph19105837 (PMC9141353; doi:10.3390/ijerph19105837)
Supplement: Supplementary file 1 [file ijerph-19-05837-s001.zip › ijerph-1697946-supplementary.pdf]

Supplementary Table S1. list of abbreviation and relative *"in extenso"* meaning.

Acronymous	Extenso
CTS	Calcific tendinopathy of the shoulder
US	Ultrasound
UGN	US-guided needling
BEN	Biocompatible Electrical Neurostimulation
NRS	Numerical Rating Scale
SPADI	Shoulder Pain and Disability Index
T0	Baseline
T1	15 days from enrollment
T2	40 days from enrollment
HLA1	Human Leukocyte Antigens 1
NSAIDs	Non-steroidal anti-inflammatory drugs
CoTreatG	Combined-treatment-group (UGN+BEN)
UGN-A	UGN-alone treatment
CER	Control group event rate
α-error	Type-I error (the mistaken rejection of an actually true null hypothesis)
β-error	Type-II (the failure to reject a null hypothesis that is actually false)
LMM	Linear Mixed Models
ρ	Intraclass coefficient correlation
R^2e	assess the proportion of within-person variation explained by time

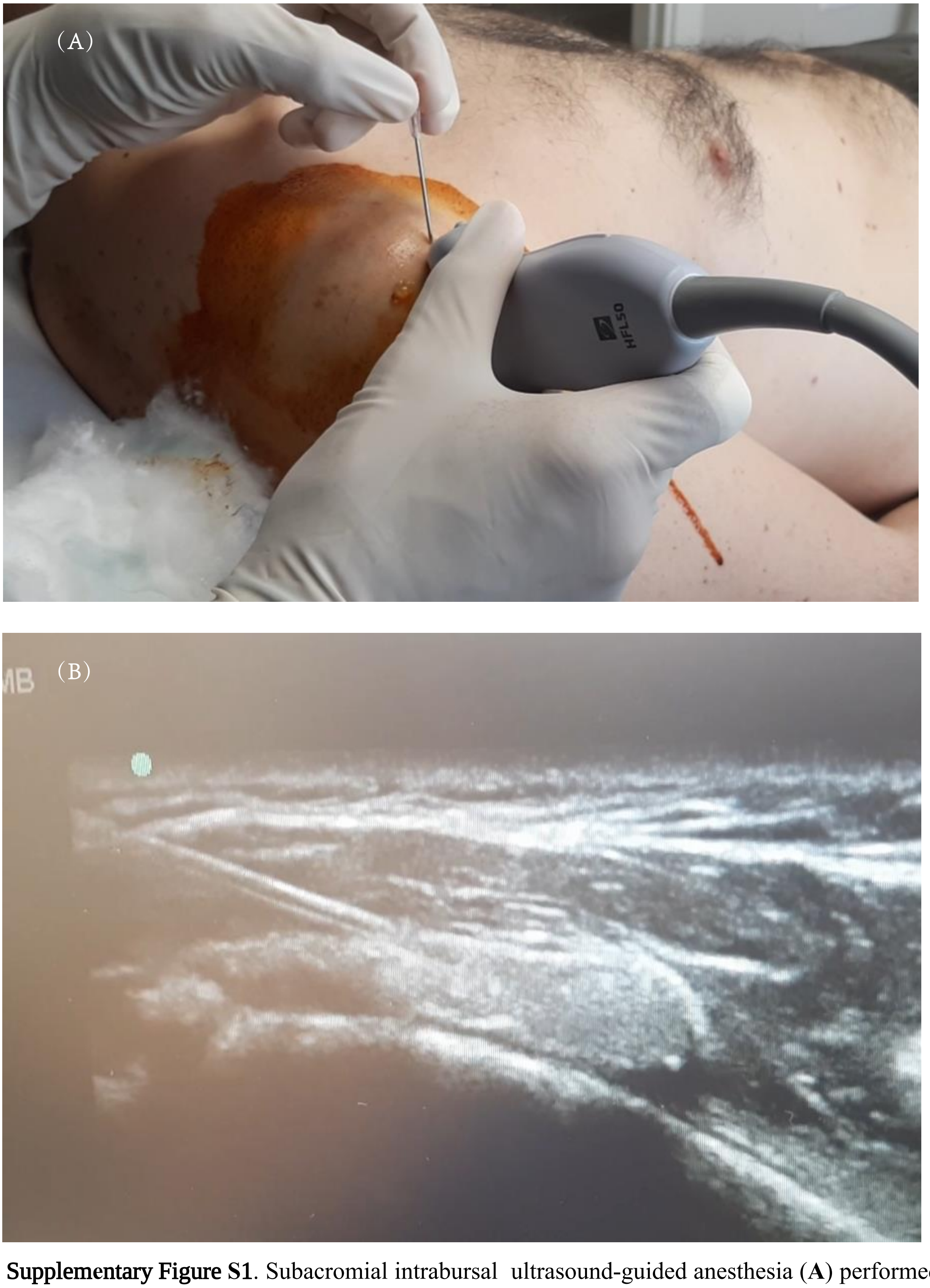

Supplementary Figure S1. Subacromial intrabursal ultrasound-guided anesthesia (**A**) performed with a 22G 40 mm needle inclined at 60° from the skin surface, with an ultrasound check (**B**) of the progression of the needle until the sub-acromial border.

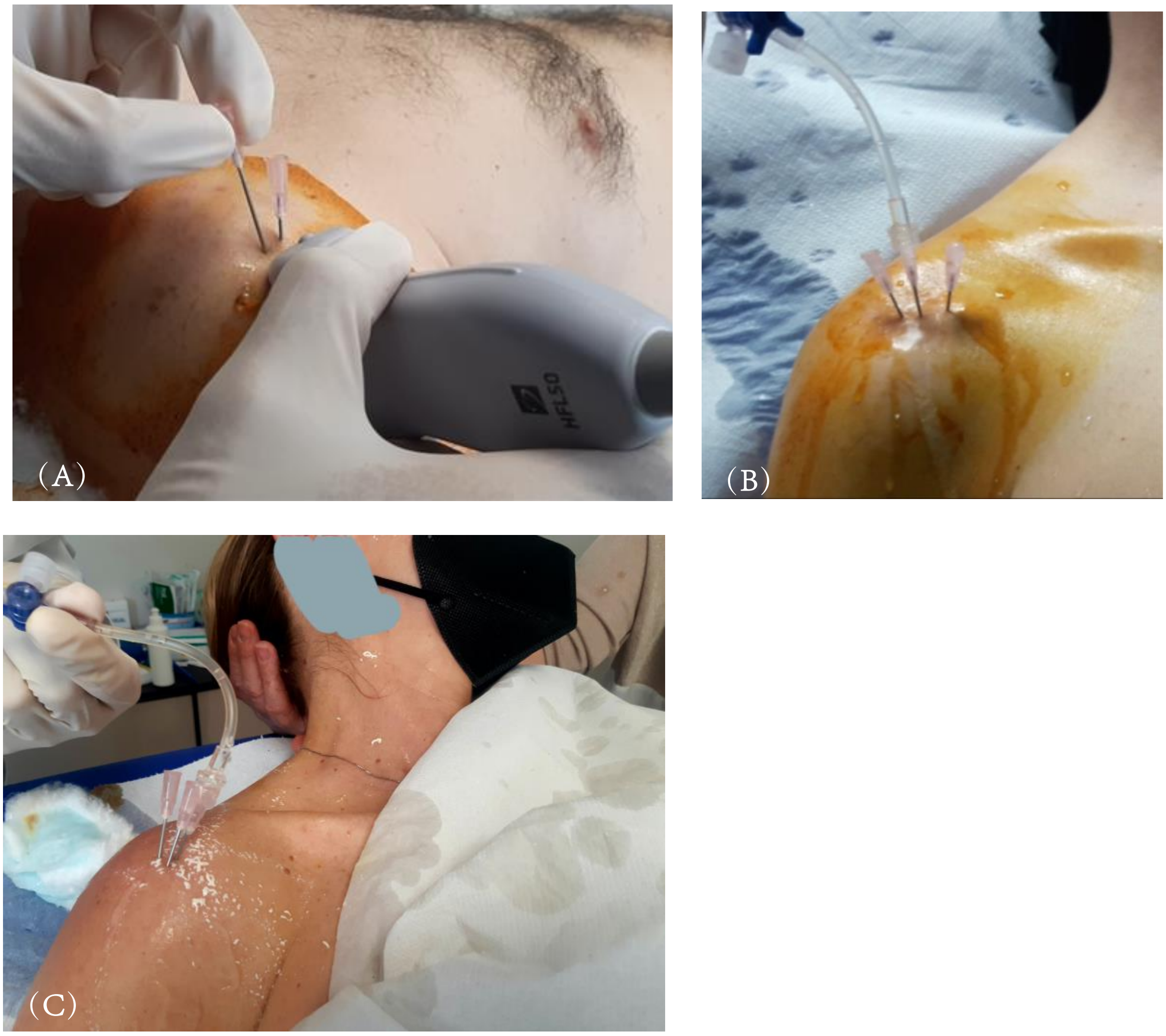

Supplementary Figure S2. (**A**): Three needles (16/18/G) were inserted, with a 60° angle of inclination from the skin surface, with the position of the needles checked by US until the intratendinous calcification was achieved. (**B**): The three 40 mm needles were placed into the calcification and they were communicating with each other. Therefore, a small connector in alternative succession to each needle was insert-ed. This connector was subsequently attached with a 30cc syringe containing saline solution. (**C**): Last phase of the procedure, with a succession of lavage and aspirations with saline and lidocaine 2% in the context of the calcification, in order to remove the calcium residues from the tendon.

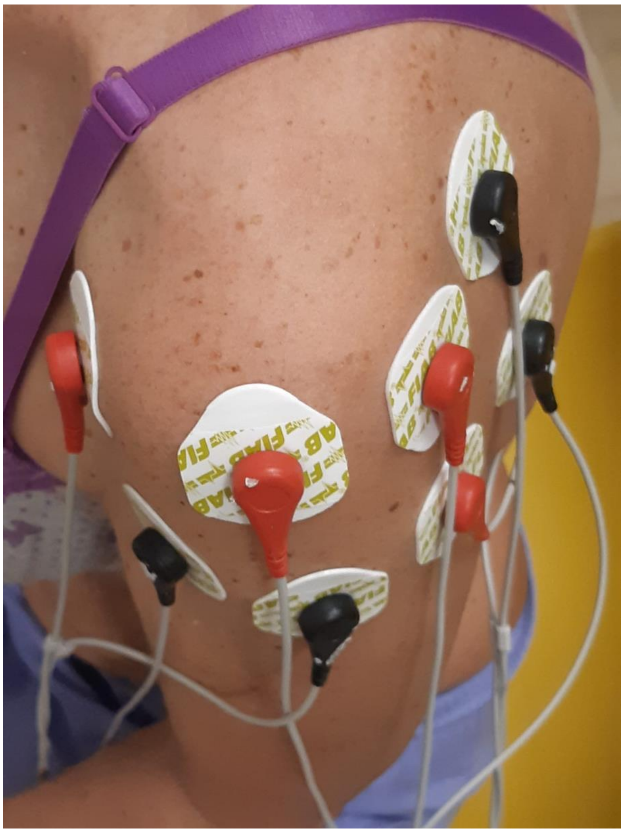

Supplementary Figure S3. The LIFE-STIM™ variable frequency electrical transcutaneous analgesic modulation device. The electrodes were positioned as follows: (1) two pairs of electrodes of channel 1 in the anterolateral region of the deltoid with the red electrodes proximally and the black electrodes more distally; (2) the first pair of electrodes of channel 2 was placed in the posterior lateral area of the deltoid, with the red electrode placed at the posterior edge of the acromion process and the black electrode placed 5cm posteriorly. The second pair of electrodes of channel 2 was placed parallel to the first pair, but 8–10 cm lower.

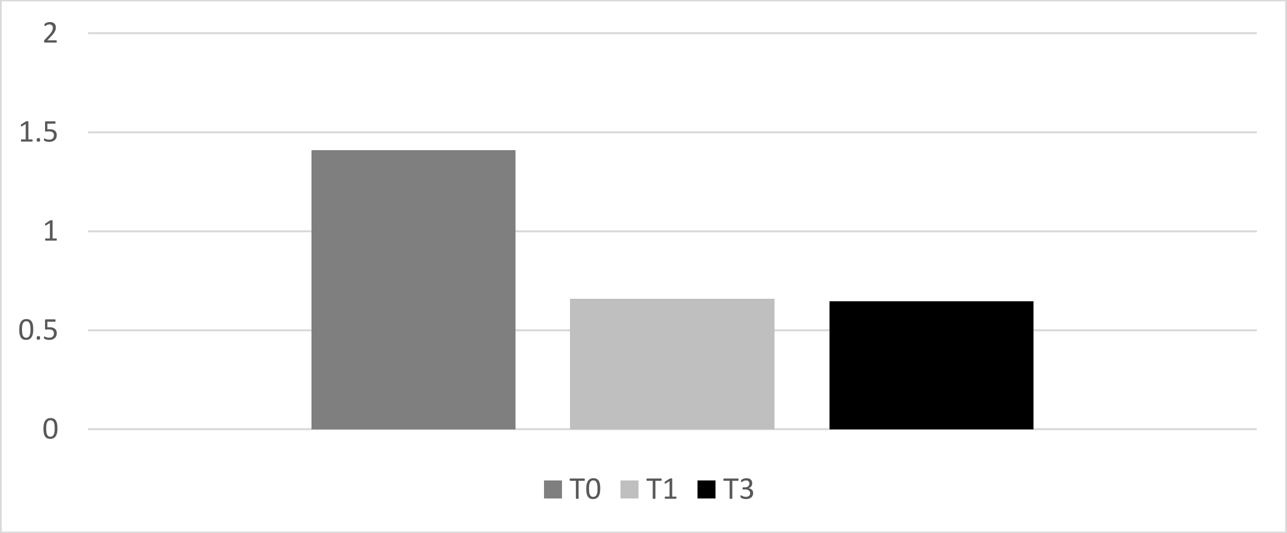

Supplementary Figure S4. Variation across times of the study in the absolute dimension (cm) of the tendon calcification in the subjects enrolled. Statistical differences among times of the study were assessed using Linear Mixed Models.
